# Phase-Controlled Cobalt Catalyst Boosting Hydrogenation of 5-Hydroxymethylfurfural to 2,5-Dimethylfuran

**DOI:** 10.3390/molecules28134918

**Published:** 2023-06-22

**Authors:** Kaixuan Yang, Naimeng Chen, Xiaomiao Guo, Ruoqi Zhang, Xiaoyu Sheng, Hui Ge, Zhiguo Zhu, Hengquan Yang, Hongying Lü

**Affiliations:** 1 Department College of Chemistry and Chemical Engineering, Yantai University, 32 Qingquan Road, Yantai 264005, China; yangkaixuanyt@ytu.edu.cn (K.Y.); 19712025872@s.ytu.edu.cn (X.S.); zhuzg@ytu.edu.cn (Z.Z.); 2 School of Chemistry and Chemical Engineering, Shanxi University, Taiyuan 030006, China; 3 State Key Laboratory of Coal Conversion, Institute of Coal Chemistry, Chinese Academy of Sciences, Taiyuan 030001, China; gehui@sxicc.ac.cn; 4 University of Chinese Academy of Sciences, Beijing 100049, China

**Keywords:** phase-controlled, temperature-induced phase transition, HCP-Co, FCC-Co, hydrogenation

## Abstract

The search for non-noble metal catalysts for chemical transformations is of paramount importance. In this study, an efficient non-noble metal catalyst for hydrogenation, hexagonal close-packed cobalt (HCP-Co), was synthesized through a simple one-step reduction of β-Co(OH)_2_ nanosheets via a temperature-induced phase transition. The obtained HCP-Co exhibited several-times-higher catalytic efficiency than its face-centered cubic cobalt (FCC-Co) counterpart in the hydrogenation of the C=C/C=O group, especially for the 5-hydroxymethylfurfural (HMF) hydrogenation (8.5-fold enhancement). Density functional theory calculations demonstrated that HMF molecules were adsorbed more firmly on the (11$\bar{2}$0) facet of HCP-Co than that on the (111) facet of FCC-Co, favoring the activation of the C=O group in the HMF molecule. The stronger adsorption on the (11$\bar{2}$0) facet of HCP-Co also led to lower activation energy than that on the (111) facet of FCC-Co, thereby resulting in high activity and selectivity. Moreover, HCP-Co exhibited outstanding catalytic stability during the hydrogenation of HMF. These results highlight the possibility of fabricating hydrogenation catalysts with satisfactory catalytic properties by precisely tuning their active crystal phase.

## 1. Introduction

Owing to the increasing consumption of fossil fuels and related environmental problems, seeking sustainable and clean fuels to replace fossil resources has become one of the most urgent research challenges [1,2]. 5-Hydroxymethylfurfural (HMF), a promising biomass-derived platform molecule for the synthesis of value-added chemicals, is available at an industrial scale via the hydrolysis−dehydration of the hemicellulose part of lignocellulosic biomass [3,4,5,6,7]. Those lignocellulose-derived HMF molecules are regarded as bridges between biomass resources and high-value-added chemicals [8,9,10]. However, HMF bears three functional constituents (C−OH, C=O, and the furan ring), which increase the difficulty of its conversion to specific chemicals, such as 2,5-dimethylfuran (DMF) [11,12], 2,5-bis(hydroxymethyl)furan (BHMF) [10], and 1,6-hexanediol [13]. The rational design of efficient catalysts for the hydrogenation of HMF has, thus, become a crucial task for these green chemical transformations [3,4,12].

Noble catalysts, such as Pd, Ru, and Pt, have long been used for the hydrogenation reactions of HMF owing to their remarkable catalytic activity and selectivity [14,15]. Studies on catalytic systems based on noble metals are achieving continuous progress toward increasing the yields of DMF. For example, Talpade et al. reported a Pd-based catalyst for DMF production, which provided a DMF yield of 96% at 150 °C within 2 h [16]. However, the noble metal catalysts suffer serious drawbacks, such as scarcity and high prices. Moreover, poor ability to withstand sulfur and CO toxicity hinders their large-scale application [10,17,18,19,20]. Completely or partially replacing noble catalysts with earth-abundant non-noble metal catalysts, such as Ni, Co, Cu, and noble–non-noble metal alloys, has been proven to be a promising and challenging strategy to address these issues [15,21]. In this context, improving the catalytic properties (activity, selectivity, and stability) of non-noble metal catalysts to match or exceed those of the well-established noble metal counterparts is still a major challenge [21,22,23]. 

Cobalt catalysts, which have the advantage of being earth-abundant, have been widely investigated and applied in hydrogenation reactions [24,25,26,27]. Kouachi et al. [24] reported that metallic Co catalysts with different supports exhibited good catalytic performances in citral hydrogenation. Cobalt catalysts with dispersed subnanometer particles were also used for the hydrogenation of nitroarenes, showing remarkable activity and selectivity [25]. A second element was incorporated into the cobalt lattice to improve the catalytic properties. A Co-based silicide intermetallic compound prepared via microwave-assisted chemical vapor deposition exhibited significantly high activity in the selective hydrogenation of phthalic anhydride [26]. Similarly, Co_4_N nanosheets showed a turnover frequency of 25.6 h^−1^ in CO_2_ hydrogenation [27]. Unfortunately, most Co-based catalysts have inferior activity to noble metal- and Ni-based catalysts. 

Metallic cobalt exists in three crystal phases, namely face-centered cubic (FCC), hexagonal closed-packed (HCP), and metastable cubic (ɛ-C), which are closely related to its catalytic properties [28,29,30,31,32]. The relationship between catalytic properties and crystal phases of cobalt-based catalysts in the FT and ammonia synthesis has been extensively studied [29,33,34,35,36]. For instance, HCP-Co exhibited higher activity than FCC-Co for the FT synthesis [33,34]. DFT (density functional theory) calculations revealed that the different exposed facets on the two catalysts accounted for the activity difference. The (10$\bar{1}$1) facet of HCP-Co nanoparticles is the active center for CO dissociation and carbon chain growth [33,34,35]. Similarly, DFT calculations showed that HCP-Co was more active than FCC-Co in the NH_3_ synthesis, owing to the exposure of (10$\bar{1}$2) and (11$\bar{2}$1) facets in HCP-Co [36]. However, very few systematic studies were performed to tune the Co crystal phase and then optimize the catalytic performances of cobalt catalysts for the hydrogenation of biomass-derived molecules. In addition, the structure–activity relationship for hydrogenation over cobalt catalysts has rarely been discussed [27,31,37]. This motivated us to develop a simple method for the precisely controllable fabrication of a specific crystal phase of cobalt to boost the hydrogenation activity and simultaneously determine the correlation between the crystal structure and hydrogenation activity of cobalt catalysts. 

Herein, we propose a one-step reduction of β-Co(OH)_2_ nanosheets strategy to synthesize an unsupported Co catalyst with a pure HCP phase. The catalytic performance of the HCP-Co catalyst in the hydrogenation of HMF was systematically investigated. The structure–performance relationship between the crystal phase of cobalt and the catalytic performance was systematically analyzed through characterizations and theoretical calculations. Moreover, the cause of the deactivation of the catalyst during the stability test was also investigated.

## 2. Results and Discussion

### 2.1. Characterization of Catalysts

**Figure 1:**
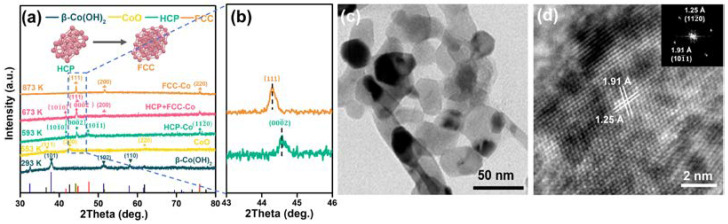
XRD patterns (**a**) and enlarged XRD patterns (**b**) of Co-based catalysts prepared with different reduction temperature. TEM image (**c**) and HRTEM image (**d**) of HCP Co reduced at 593 K. The inset image corresponds to HCP Co-associated fast Fourier transformation.

The preparation of the cobalt catalysts started with the synthesis of β-Co(OH)_2_ nanosheets through a simple precipitation method [27]. The as-obtained β-Co(OH)_2_ nanosheets were heated to the specific temperature and maintained for 2 h in a H_2_/Ar mixture atmosphere, forming cobalt catalysts with different crystal phases [38]. The crystal phase transformation of β-Co(OH)_2_ nanosheets during the temperature-induced phase transition (TIPT) process is shown in Figure 1a. Diffraction peaks were observed at 2θ = 37.6, 51.1, and 57.6°, corresponding to the (101), (102), and (110) planes of the β-Co(OH)_2_ precursor, respectively. The diffraction pattern of the transition state reduced at 553 K displayed three broad peaks at 36.5, 42.4, and 61.5°, corresponding to the (111), (200), and (220) planes of CoO (JCPD No.78-0431), respectively. When the reduction temperature increased to 593 K, the peaks of CoO disappeared and metallic Co was detected. The XRD pattern of the sample reduced at 593 K displayed four peaks at 41.6, 44.7, 47.6, and 61.5°, corresponding to the (10$\bar{1}$0), (00$\bar{0}$2), (10$\bar{1}$1), and (11$\bar{2}$0) planes of HCP-Co (JCPD No. 05-0727), respectively. A peak at 44.2°, corresponding to the (111) plane of FCC-Co, appeared when the reduction temperature exceeded 673 K. This indicated the formation of composite HCP-Co/FCC-Co phases. After treatment at 873 K in a H_2_/Ar mixture, all peaks belonging to HCP-Co disappeared, along with the appearance of peaks at 44.2, 51.5, and 75.8°, corresponding to the (111), (200), and (220) planes of FCC-Co (JCPD No. 15-0806). The crystallite size of HCP-Co, calculated using the Scherrer equation, was 28.2 nm (Table S1), similar to that in Figure 1c. The calculated crystallite size of FCC-Co was larger than that of HCP-Co, as a result of the high-temperature reduction. The HRTEM image in Figure 1d shows that the prepared HCP-Co catalyst exhibited lattice fringes with interplanar spacings of 1.25 and 1.91 Å, indexed to the (11$\bar{2}$0) and (10$\bar{1}$1) planes, respectively. In contrast, as shown in Figure S1, the FCC-Co sample exposed the (111) crystalline plane after being reduced at 873 K. This is in good agreement with the XRD results, showing that the crystal phase changed from HCP-Co to FCC-Co with increasing reduction temperature.

**Figure 2:**
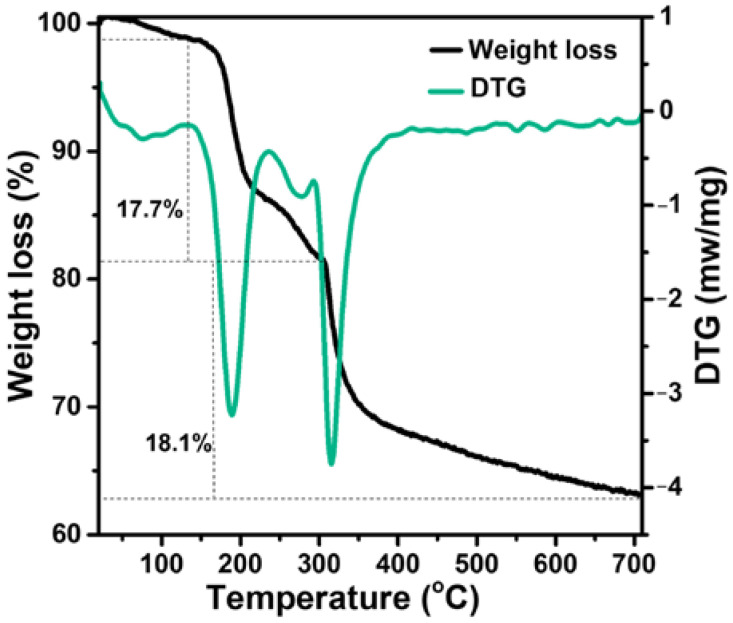
Thermal gravity profiles of the β-Co(OH)_2_ nanosheets under H_2_ atmosphere.

The TIPT process of β-Co(OH)_2_ was also investigated using thermogravimetric (TG) analysis under an H_2_/Ar atmosphere (10% H_2_). As shown in Figure 2, the mass loss below 405 K was attributed to the elimination of water adsorbed on the surface of the nanosheets. The thermal decomposition of β-Co(OH)_2_ to CoO was responsible for the 17.7% mass loss between 405 and 583 K, whereas the reduction of CoO to Co was responsible for the mass loss between 583 and 973 K. The total weight loss from 405 to 973 K was 35.8%, close to the theoretical weight loss of the transformation from β-Co(OH)_2_ to Co (36.6%). The XRD and TG results of the TIPT process of β-Co(OH)_2_ nanosheets reveal the occurrence of a sequence of crystalline phase transformations during the reduction process: β-Co(OH)_2_ → CoO → HCP-Co → HCP-FCC-Co → FCC-Co. It indicates that the TIPT method allows the Co phase to be precisely controlled by varying the reduction temperature [31]. In addition, it is worth noting that the different crystal phases of cobalt exhibited various exposed facets, likely affecting the catalytic properties of the cobalt catalysts [29].

### 2.2. Hydrogenation Reaction over Cobalt Catalysts

**Scheme 1:**
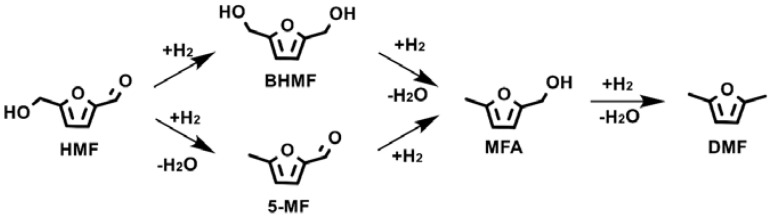
Proposed reaction pathway of HMF hydrogenation.

**Figure 3:**
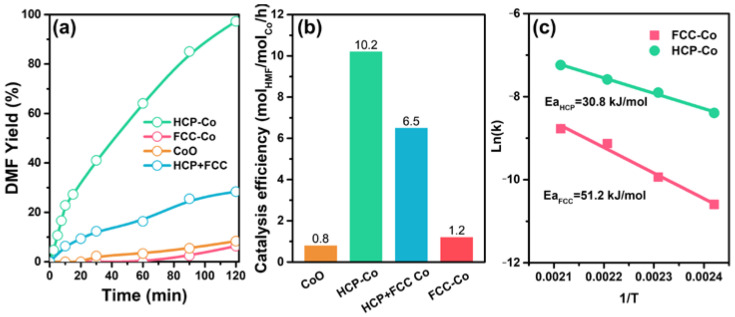
(**a**) Hydrogenation performance of various Co catalysts. (**b**) CE as a function of the crystal phase of Co catalysts. CE is defined as the moles of the converted reactant per mole of catalyst per h (mol_HMF_ ·mol_Co_^−1^ ·h^−1^). (**c**) The Arrhenius plots and calculated apparent activation energy. Reaction conditions: HMF, 0.378 g; HCP-Co, 0.05 g; 30 mL THF; agitation speed, 1000 rpm; 453 K; 2 MPa.

The hydrogenation of the biomass platform molecule HMF was selected to test the catalytic properties of the as-prepared cobalt catalysts. The functional groups (C=O, C=C, C–O, and furan ring) of the HMF molecule make it suitable for further hydrogenation conversions. As shown in Scheme 1, the main products of HMF hydrogenation could be BHMF, 5-methylfurfural (5-MF), 5-methylfurfurylalcohol (MFA), and the final product DMF. The hydrogenation of HMF is a cascade of reactions, and the selectivity to DMF strongly depends on the HMF conversion. The results of HMF hydrogenation over the as-prepared CoO, HCP-Co, FCC-Co, and mixed-phase Co (with dual HCP-Co and FCC-Co) catalysts are displayed in Figure 3 and Figure S2. As shown in Figure S2, the CoO catalyst exhibited inferior catalytic activity and selectivity to DMF (<20%) due to the absence of the metallic phase. In contrast, the HCP-Co catalyst showed outstanding catalytic performance in the hydrogenation of HMF to DMF, with a 97.3% DMF yield under 100% HMF conversion (Figure 3a and Figure S2). In addition, the cobalt catalyst obtained whilst using a higher reduction temperature, with mixed HCP-Co/FCC-Co phases, exhibited poor catalytic activity and selectivity compared to HCP-Co. FCC-Co exhibited inferior activity and the lowest DMF yield (6.4%) for HMF hydrogenation. It is worth noting that the activity of the as-prepared catalysts is strongly dependent on the crystal phase of cobalt. Similarly, as shown in Figure 3b, the cobalt catalyst with pure HCP phase exhibited the maximum CE (10.2 mol_HMF_·mol_Co_^−1^·h^−1^), whereas a lower CE was observed over HCP-FCC-Co. Furthermore, the HCP-Co catalyst showed an 8.5-fold increase in CE compared to its counterpart FCC-Co (1.2 mol_HMF_·mol_Co_^−1^·h^−1^). It indicated that FCC-Co was less active for HMF hydrogenation, and increasing the proportion of the FCC-Co phase in the Co catalysts would cause a decrease in the activity for HMF hydrogenation. These results strongly confirm the key role of the Co phases in the hydrogenation of HMF to DMF. Furthermore, the apparent activation energies (Ea) for HMF hydrogenation over HCP-Co and FCC-Co were also calculated (see details in Figure S3 and Table S2). As shown in Figure 3c, the Ea for HMF hydrogenation over HCP-Co was 30.8 kJ/mol, much lower than that over FCC-Co (51.2 kJ/mol). Lyu et al. reported that the activation barrier for CO dissociation on the (11$\bar{2}$0) facet of HCP-Co (1.39 eV) was ca. 1.09 eV lower than that on the dominating (111) facet of the FCC-Co crystal phase (2.48 eV) [33]. Similarly, the calculated reaction rate of ammonia synthesis on the (11$\bar{2}$0) facet was much higher than that on the (111) facet of FCC-Co [36]. Therefore, the difference in the catalytic performances of the HCP-Co and FCC-Co catalysts observed in this study could be ascribed to the different crystal faces exposed on their crystalline phases, which led to various HMF hydrogenation activities.

As shown in Table 1, the surface area of the cobalt catalysts decreased with increasing reduction temperature. It is generally accepted that the surface area affects the rate of mass transfer and consequently the activity of the catalysts. CNTs-supported HCP-Co and FCC-Co catalysts were prepared using a similar method to investigate the effect of the surface area. As shown in Figure S4a and Table S3, the HCP-Co/CNTs and FCC-Co/CNTs catalysts had similar surface areas. Furthermore, the peaks in the XRD patterns in Figure S4b corresponded to the HCP and FCC phases. The two supported cobalt catalysts were applied in the hydrogenation of HMF, and the results are shown in Figure S5. The HMF conversion over HCP-Co/CNTs was higher than that over FCC-Co/CNTs. Furthermore, the DMF yield over HCP-Co/CNTs after 6 h of reaction was approximately three times higher than that over FCC-Co/CNTs. The results of all reaction tests carried out over the supported cobalt catalysts indicated that the superior catalytic performance of the HCP-Co catalyst could be ascribed to the HCP crystal phase.

**Figure 4:**
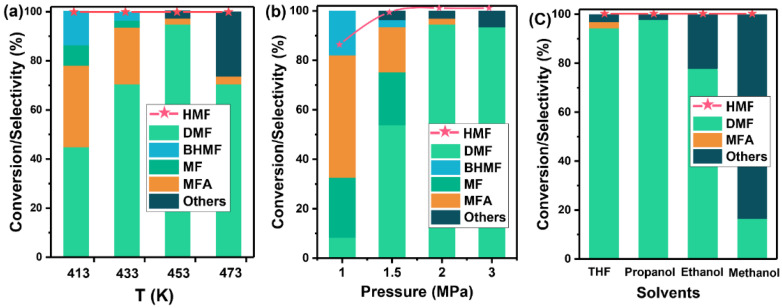
Effect of reaction temperature (2 MPa, THF) (**a**), H_2_ pressure (453 K, THF) (**b**), and solvents (**c**) on HMF hydrogenation over the -Co catalyst (2 MPa, 453 K). Reaction conditions: HMF, 0.378 g; HCP-Co, 0.05 g; agitation speed, 1000 rpm.

Further tests were conducted to evaluate the catalytic performance of the HCP-Co catalyst in the hydrogenation of HMF to DMF. As shown in Figure 4a, 100% HMF conversions were obtained over the HCP-Co catalyst at all different temperatures, while the DMF selectivities varied; in particular, the latter were only 44.5% and 71.5% at 413 and 433 K, respectively. With increasing temperature, a maximum DMF yield of 97.3% was obtained at 453 K. However, a higher reaction temperature (473 K) leads to a decrease in DMF selectivity (71.0%), owing to the formation of humins (as discussed along with the TG characterization results below). As shown in Figure 4b, the conversion of HMF increased from 83.2% at 1 MPa to 100% at 2.0 MPa, leading to the DMF yield increasing from 8.9% to 97.3%. Upon further increasing the reaction pressure to 3 MPa, the DMF yield slightly decreased to 93.3% due to the formation of excessive hydrogenation products. In addition, different solvents resulted in significant changes in the selectivity to DMF over the HCP-Co catalyst (Figure 4c). The DMF selectivity was >95% in THF and 2-propanol but ca. 80% selectivity in ethanol and less than 20% in methanol. The low selectivity to DMF in ethanol and methanol could be attributed to the etherification reaction to form ethers [39,40].

Furthermore, the performance of HCP-Co in the HMF hydrogenation to DMF is compared with that of various established catalysts in Table S4 [41,42,43,44,45,46,47,48]. The catalytic performance of the HCP-Co catalyst is comparable to that of precious metal catalysts and superior to that of non-noble metallic catalysts under similar conditions. These results confirm the excellent performance of the as-prepared HCP-Co catalyst, which can, thus, be used as an alternative to precious metals for HMF conversion.

To investigate the general applicability of HCP-Co, the present cobalt catalysts were also applied to the hydrogenation of other substrates with unsaturated functional groups (Figures S6–S10). As shown in Table 2, HCP-Co possessed a 10-fold higher activity than FCC-Co in the hydrogenation of cinnamyl aldehyde. Moreover, the HCP-Co catalyst also exhibited superior catalytic performance in the hydrogenation of acetophenone, styrene, phenol, and nitrobenzene, compared with the FCC-Co catalysts. In particular, the HCP-Co catalyst showed a much higher CE than FCC-Co in these industrially important hydrogenation reactions of unsaturated substrates.

**Figure 5:**
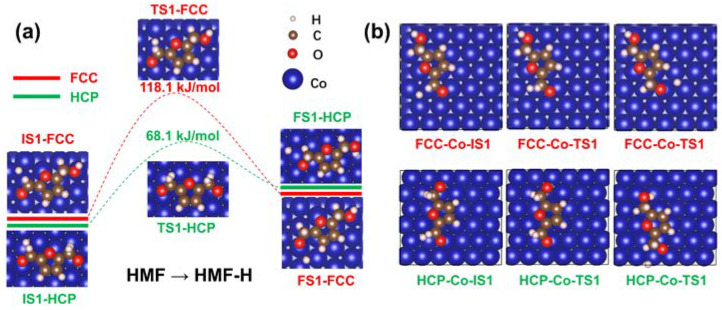
(**a**) Reaction potential energy of HMF + H_2_→BHMF reaction on the HCP-Co (11$\bar{2}$0) and FCC-Co (111) surfaces. (**b**) Optimized configurations of the initial states (IS), transition states (TS), and final states (FS) for the HMF + H_2_→BHMF reaction on HCP-Co (11$\bar{2}$0) and FCC-Co (111) surfaces. C, O, H, and Co atoms are shown in grey, red, white, and blue balls, respectively.

According to the DFT calculations, the (11$\bar{2}$0), (10$\bar{1}$2), and (10$\bar{1}$0) planes dominated the HCP-Co Wulff shape, whereas the (111) plane occupied most of the surface of FCC-Co [35]. Therefore, to clarify the significant difference in the hydrogenation performance of these Co catalysts with different phases, DFT calculations were performed to investigate the reaction energy barriers for the first step of HMF hydrogenation on HCP-Co (11$\bar{2}$0) and FCC-Co (111) facets [49]. In the initial state, HMF is adsorbed on the surface of the Co site, and the two H atoms from the H_2_ dissociation are adsorbed nearby. According to a previous study, the two dissociated H atoms tend to hydrogenate the first carbonyl carbon and then the carbonyl oxygen atom [50]. The potential changes along the reaction coordinate on the HCP-Co and FCC-Co surfaces are shown in Figure 5a. The transition state (TS) barrier of HMF hydrogenation on the surface of HCP-Co (11$\bar{2}$0) was 68.1 kJ/mol, which was much lower than that on the surface of FCC-Co (111). Therefore, the lower energy barrier of the first step of HMF hydrogenation on the HCP-Co surface is contributed to its high activity in the HMF hydrogenation.

To investigate the significant difference in reaction barriers between the two cobalt phases, we analyzed the adsorption energies of HMF and the Co-O/C=O bond lengths in all initial, transition, and final states (Figure 5b and Tables S5 and S6). The HMF adsorption energy on HCP-Co was much higher than that on FCC-Co (−1.71 vs. −1.17 eV). This strong adsorption provides sufficient activation of the C=O double bond (C=O bond length: 1.303 Å for HCP-Co vs. 1.343 Å for FCC-Co), reducing the hydrogenation barrier. Moreover, the HMF and HMF-H on HCP-Co bond to the surface Co atom with an O-Co bonding length of 1.859 and 1.814 Å. However, the weak adsorption of the HMF and HMF-H on the FCC-Co surface leads to longer Co-O bond lengths (2.004 and 1.835 Å), which results in a much higher reaction barrier. All the DFT calculations revealed that the crystal phase controls the catalytic properties of cobalt catalysts in the HMF hydrogenation.

**Figure 6:**
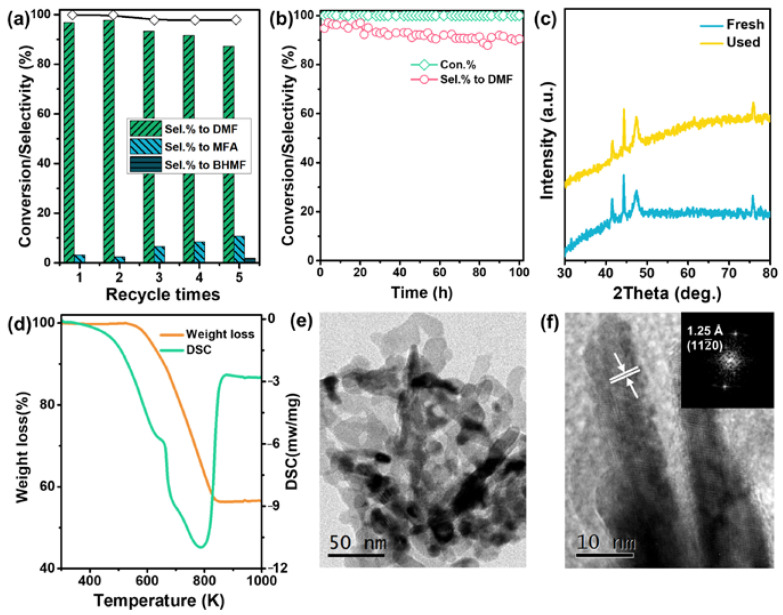
Stability test of HCP-Co on HMF hydrogenation in batch reactor (**a**) and fixed-bed reactor (**b**). (**c**) XRD patterns; (**d**) TG–DTG curves; (**e**) TEM images of used HCP-Co catalyst after 100 h test; (**f**) HRTEM images of used HCP-Co catalyst after 100 h test. The inset image corresponds to HCP Co-associated fast Fourier transformation. Reaction conditions: batch reactor, 0.378 g HMF, 0.05 g catalyst, 2 MPa, 453 K, 1000 rpm; fixed-bed reactor, 0.05 g catalyst, 2 MPa, 453 K, contract time 2 h, and H_2_/substrate = 300.

The recyclability and stability of the HCP-Co catalyst were tested using a batch and a fixed-bed reactor, respectively. As shown in Figure 6a, only a 3% reduction in HMF conversion was observed after a five-times cycle test in the batch reactor. At the same time, the selectivity to DMF changed from 97% to 87%. A similar trend was observed when HCP-Co was used in the fixed-bed reactor (Figure 6b). The conversion of HMF remained at 99%, while the DMF selectivity showed a slight decrease. The used HCP-Co catalyst was characterized to uncover the reason for the decrease in catalytic capacity. As shown in Figure 6c, the used catalyst maintained the HCP phase, and the calculated particle size was similar to that of the fresh HCP-Co catalyst (Table S1). TG analysis was conducted to examine the carbon deposition on the spent HCP-Co catalyst after a 100 h test. As shown in Figure 6d, a sharp weight decline was observed at around 788 K in the TG–DTG curve, resulting from the formation of humins (polymerization of HMF) and carbon deposition [12]. Similarly, as shown in Figure 6f, an amorphous layer covered the surface of HCP-Co. The deposited carbonaceous material may deactivate the catalyst by covering the active sites or through pore blocking [51,52]. It indicated that the activity loss could be ascribed to the carbon deposition on the surface of HCP-Co. Moreover, the morphology of the spent catalyst appeared to be slightly different from that before the reaction (Figure 1c). The change in the morphology of the used cobalt catalyst would also result in deactivation. Therefore, the difference in the morphology HCP-Co could be another cause for its deactivation during the stability test.

## 3. Materials and Methods

### 3.1. Materials

The following chemicals were utilized as provided without further purification: Co(NO_3_)_2_·6H_2_O (99%, Sinopharm Chemical Reagent Co., Ltd., Beijing, China), NaOH (≥98%, Shanghai Aladdin Bio-Chem Technology Co., Ltd., Shanghai, China), 5-hydroxymethylfurfural (HMF, 98%, Shanghai Bide Pharmatech Co., Ltd., Shanghai, China), 2,5-dimethylfuran (DMF, 99%, Shanghai Energy Chemical Co., Ltd., Shanghai, China), tetrahydrofuran (THF, 99%, Shanghai Macklin Biochemical Co., Ltd., Shanghai, China), 2,5-bis(hydroxymethyl)furan (BHMF, 98%, Shanghai Macklin Biochemical Co., Ltd., Shanghai, China), 2-methyl-5-hydro-xymethylfuran (MFA, 99%, Shanghai Sigma-Aldrich Co., Ltd., Shanghai, China), Cinnamyl aldehyde (98%, Shanghai Macklin Biochemical Co., Ltd., Shanghai, China), Acetophenone (≥98%, Shanghai Macklin Biochemical Co., Ltd., Shanghai, China), Styrene (99%, Shanghai Macklin Biochemical Co., Ltd., Shanghai, China), and Phenol (≥99.5%, Shanghai Aladdin Bio-Chem Technology Co., Ltd., Shanghai, China).

### 3.2. Catalyst Preparation

The hexagonal β-Co(OH)_2_ was prepared using a simple precipitation method [27]. A total of 3.0 g Co(NO_3_)_2_·6H_2_O was dissolved in 50 mL water to achieve a homogeneous solution using a magnetic stirrer. A total of 2.0 mol/L NaOH was added to the Co(NO_3_)_2_ solution dropwise. The mixture was filtered and washed with distilled water, and the formed Co(OH)_2_ was collected and dried at 333 K under a vacuum overnight.

The two-dimensional HCP-Co was synthesized as follows: β-Co(OH)_2_ was placed in a quartz tube and heated to 593 K at a rate of 10 K/min under 10% Ar/H_2_ gas flow (1 bar, 60 sccm). After keeping at 593 K for two hours, the sample was passivated using a 1% O_2_/N_2_ mixture gas flow (1 bar, 40 sccm) for 3 h at room temperature. Then, the HCP-Co catalyst was obtained. The FCC-Co and other catalysts were obtained by using different reduction temperatures.

### 3.3. Characterizations

The crystal structure of the sample was recorded by using X-ray diffraction (XRD Rigaku, Smart Lab3) with Cu Ka radiation (40 mA, 45 kV). The Brunauer–Emmett–Teller (BET) surface areas of different cobalt catalysts were measured via N_2_ adsorption-desorption experiments performed on Micromeritics ASAP2020 at liquid nitrogen temperature. Transmission electron microscope (TEM) images were obtained using TECNAI 20S-TWIN. Thermo Gravimetric Analysis (TGA) was performed using a NETZSH STA409PC instrument (NETZSH GERATEBAU GmbH Germany, Selb, Germany), and the experiment was carried out in an air or 10% H_2_-Ar mixture atmosphere.

### 3.4. Catalytic Testing

HMF, cinnamyl aldehyde, acetophenone, styrene, and phenol were used for catalytic testing. HMF hydrogenation, for instance, was carried out in a 50 mL stainless-steel autoclave (Anhui Kemi Machinery Technology Co., Ltd., Hefei, China). A mixture of HMF (0.378 g), THF (30 mL), tetradecane (internal standard), and catalyst (0.05 g) was put in the sealed reactor equipped with a magnetic stirring bar. After replacing it with 1 MPa H_2_, the reactor was pressurized to 2 MPa H_2_ and then heated to a given temperature. After the reaction, the reactor was cooled to room temperature. The products were collected and analyzed using GC-MS (7890B-5977B) and quantitatively determined using an Agilent 7890B GC (HP-5 column, FID detector). The conversion, selectivity, yield, and catalysis efficiency (CE) are defined as follows:

(1) $$Conversion=\frac{n_{HMF,initial}-n_{HMF,final}}{n_{HMF,initial}}\times 100\%,$$

(2) $$Selectivity=\frac{n_{DMF}}{n_{HMF,initial}-n_{HMF,final}}\times 100\%,$$

(3) $$Yield=\frac{n_{DMF}}{n_{HMF,initial}}\times 100\%,$$

(4) $$CE=\frac{n_{HMF,initial}-n_{HMF,t}}{n_{Co}\cdot t}\;({\mathrm{mol}}_{\mathrm{HMF}}\;\cdot {\mathrm{mol}}_{\mathrm{Co}}^{-1}\;\cdot \;h^{-1}).$$

The kinetic analysis of the HMF hydrogenation reaction was conducted using varied conversions of HMF by changing the reaction time. Firstly, it is assumed that the hydrogenation reaction of HMF under low conversion is a first order reaction. Different rotate speeds were tested, and 1000 r/min was used to eliminate the mass transfer effects. The following equation represented the pseudo-first-order model for the hydrogenation of HMF. The k of different catalysts was calculated using the following Equation (5):

(5) $$-ln(1-X)=k_{HMF}\cdot \tau .$$

The essential linear relationship of −ln(1 − X) and τ is shown in supporting information (Figure S3) for the data obtained from the hydrogenation of the HMF reaction. The linear relationships illustrate that the overall reaction is pseudo-first-order.

### 3.5. Theoretical Method

The total energies and atomic structure were calculated using spin-polarized density functional theory (DFT) with an exchange–correlation potential in generalized gradient approximation (GGA). All calculations were performed using the Perdew–Burke–Ernzerhof (PBE) form of the GGA functional implemented in the Vienna Ab Initio Simulation Package (VASP) [53,54]. The cutoff energy of the plane–wave basis was set at 400 eV. The Co surface structure with periodic boundary conditions was modeled using a large supercell of 12.44 × 7.46 × 25 Å^3^ for the Co surface of FCC (111) and 12 × 13 × 25 Å^3^ for the Co surface of HCP (11$\bar{2}$0), eliminating the artificial interaction between the surface in adjacent unit cells. The convergence thresholds for structural optimization and transition state (TS) search were set at 10^−4^ eV in energy and 0.05 eV/Å in force, respectively. The Brillouin zone integrations were performed using a Monkhorst–Pack 2×4×1 grid for the FCC-Co (111) catalyst and 2 × 2 × 1 grid for the HCP-Co (11$\bar{2}$0) [54].

As for FCC-Co (111), to achieve the best compromise between computational time and accuracy of the model, a four-layer slab separated in the (111) direction by a vacuum region was chosen. The vacuum distance of 15 Å was enough to avoid spurious interaction between periodic configurations. The molecule surface was optimized, allowing for the relaxation of the first two layers of the metal slab until a 1× 10^−5^ eV convergence in the total energy was reached; the three remaining layers (bulk-like) were kept fixed. As for HCP-Co (11$\bar{2}$0), a five-layer slab separated in the (11$\bar{2}$0) direction by a 15 Å vacuum region. The first two layers were relaxed and the left remaining layers were kept fixed. The climbing image nudged elastic band (CI-NEB) method was used to search for the transition states (TSs), and the minimum energy path (MEP) was constructed accordingly [55]. The van der Waals (vdW) dispersion was included using the D3 method of Gimmel [56]. To ensure accuracy, four images were used in each NEB calculation. The highest image along the MEP was denoted as the transition state. The energy barrier Ea of each elementary reaction was calculated using the energy difference between the transition state and the initial state. The corresponding reaction energy ΔE was calculated using the energy difference between the final and the initial states. By convention, positive Ea and ΔE indicate an endothermic process, while negative values correspond to an exothermic process.

The adsorption energy (E_ads_) of HMF doped in the substrate can be obtained from E_ads_ = E_M@substrate_-E_M_-E_substrate_, where E_M@substrate_ is the energy of the optimized structure of a molecular doped on the surface of Co, and E_M_ and E_substrate_ are the energies of an isolated molecular and the substrate, respectively. The negative Eads value corresponds to the exothermic process, which indicates that the embedment of metal atoms in substrates or the adsorption of gas molecules on the surface of the catalysts is energetically favorable.

## 4. Conclusions

In summary, a highly efficient HCP-Co catalyst for the hydrogenation of HMF to DMF was synthesized successfully. The catalyst was easily prepared through a one-step reduction of β-Co(OH)_2_ nanosheets via TIPT. The obtained HCP-Co catalyst exhibited superior performance for the hydrogenation of HMF and other unsaturated functional groups (C=O, C=C, and nitryl) compared to its counterpart FCC-Co. In combination with DFT calculations, it was confirmed that the exposed crystal facets controlled the catalytic properties of the cobalt catalysts. The HCP-Co catalyst exhibited a hydrogenation activity similar to those reported in precious metals, highlighting its potential for practical applications. Therefore, the present investigation provides a new strategy for the rational design of biomass-conversion catalysts with superior activity and stability by precisely tuning their active phase.

## Figures and Tables

**Table 1:** Physicochemical properties of Co-based catalysts.

Catalysts	S_BET_ (m^2^/g)	Pore Volume (cm^3^/g)	Pore Size (nm)
HCP-Co	9.95	0.113	44.77
HCP-FCC-Co	5.27	0.018	47.54
FCC-Co	3.18	0.007	46.59

**Table 2:** Hydrogenation reaction results of reactant with different unsaturated functional group over HCP-Co and FCC-Co catalysts.

Reactant	Functional Group	Reaction Conditions	Conversion (%)		Catalysis Efficiency (mol_Reactant_·mol_Co_^−1^·h^−1^) ^a^		CE_HCP-Co_/CE_FCC-Co_
			HCP-Co	FCC-Co	HCP-Co	FCC-Co	
HMF	Furyl-C=O	453 K, 2 MPa, 0.5 h, 3 mmol	87.6	19.4	10.2	1.2	8.5
Cinnamyl aldehyde	Alkylene-C=O	373 K, 2 MPa, 8 h, 4 mmol	91.6	10.8	0.8	0.09	8.9
Acetophenone	Aryl-C=O	393 K, 2 MPa, 1 h, 4 mmol	51.2	6.5	0.8	0.3	2.7
Styrene	Aryl-C=C	373 K, 2 MPa, 2 h, 4 mmol	93.8	23.5	3.4	0.7	4.9
Phenol	Aryl-C=C	453 K, 2 MPa, 2 h, 4 mmol	77.3	39.4	5.2	2.1	2.5
Nitrobenzene	Aryl-NO_2_	393 K, 2 MPa, 1 h, 4 mmol	85.8	15.0	3.5	0.3	11.7

^a^ The conversion for CE calculation was less than 10%.

## Data Availability

The data presented in this study are available on request from the author.

## Supplementary Materials

The following supporting information can be downloaded at https://www.mdpi.com/article/10.3390/molecules28134918/s1. Table S1. Physicochemical properties of Co-based catalysts; Table S2. Fitting parameters in the pseudo-first-order kinetic analysis of the hydrogenation of HMF over HCP-Co and FCC-Co; Table S3. Physicochemical properties of supported Co catalysts; Table S4. The hydrogenolysis performance of HMF to DMF over different catalysts; Table S5. The adsorption energy (eV) of HMF and BHMF on catalysts; Table S6. Calculated Co-O/C=O bond lengths in all initial, transition, and final states; Figure S1. TEM image (a) and HRTEM image (b) of FCC Co reduced at 873K. The inset image corresponds to FCC Co-associated fast Fourier transformation; Figure S2. HMF conversion (a) and DMF selectivity (b) over various Co catalysts. Reaction conditions: HMF, 0.378 g; catalyst, 0.05 g; 30 mL THF; agitation speed, 1000 rpm; 453 K; 2 MPa; Figure S3. Fitting of the pseudo-first-order kinetic model to the hydrogenation of HMF reaction experimental data collected over HCP-Co (a) and FCC-Co (b); Figure S4. N2 adsorption–desorption isotherms (a), XRD patterns (b), and enlarged XRD patterns (c) of HCP-Co/CNTs and FCC-Co/CNTs; Figure S5. Hydrogenation performance: (a) HCP-Co/CNTs; (b) FCC-Co/CNTs. Reaction conditions: HMF, 0.378 g; catalyst, 0.05 g; 30 mL THF; agitation speed, 1000 rpm; 453 K; 2 MPa; Figure S6. Cinnamyl aldehyde conversion over HCP-Co and FCC-Co catalysts; Figure S7. Acetophenone conversion over HCP-Co and FCC-Co catalysts; Figure S8. Styrene conversion over HCP-Co and FCC-Co catalysts; Figure S9. Phenol conversion over HCP-Co and FCC-Co catalysts; Figure S10. Nitrobenzene conversion over HCP-Co and FCC-Co catalysts. References [41,42,43,44,45,46,47,48] are cited in the Supplementary Materials.

## Sample Availability

Samples of the compounds are available from the authors.
