# Supplementary material for: Phase-Controlled Cobalt Catalyst Boosting Hydrogenation of 5-Hydroxymethylfurfural to 2,5-Dimethylfuran
Source: Molecules. 2023 Jun 22;28(13):4918. doi: 10.3390/molecules28134918 (PMC10343655; doi:10.3390/molecules28134918)
Supplement: Supplementary file 1 [file molecules-28-04918-s001.zip › molecules-2464465-supplementary.pdf]

Supporting Information
For
Phase-controlled Cobalt Catalyst Boosting Hydrogenation of 5-Hydroxymethylfurfural to 2,5-Dimethylfuran

Kaixuan Yang [1], Naimeng Chen [1], Xiaomiao Guo [2], Ruoqi Zhang [1], Xiaoyu Sheng [1], Hui Ge [3,4], Zhiguo Zhu [1], Hengquan Yang [2,*] and Hongying Lü [1,*]

1 Department College of Chemistry and Chemical Engineering, Yantai University, 32 Qingquan Road, Yantai 264005, China; yangkaixuanyt@ytu.edu.cn (K.Y.); 19712025872@s.ytu.edu.cn (X.S.); zhuzg@ytu.edu.cn (Z.Z.)

2 School of Chemistry and Chemical Engineering, Shanxi University, Taiyuan 030006, China

3 State Key Laboratory of Coal Conversion, Institute of Coal Chemistry, Chinese Academy of Sciences, Taiyuan 030001, China; gehui@sxicc.ac.cn

4 University of Chinese Academy of Sciences, Beijing 100049, China

* Correspondence: hqyang@sxu.edu.cn (H.Y.); hylv@ytu.edu.cn (H.L.)

Table of contents

Table S1. Physicochemical properties of Co based catalysts.

Table S2. Fitting parameters in the pseudo-first-order kinetic analysis of the hydrogenation of HMF over HCP-Co and FCC-Co.

Table S3. Physicochemical properties of supported Co catalysts.

Table S4. The hydrogenolysis performance of HMF to DMF over different catalysts.

Table S5. The adsorption energy (eV) of HMF and BHMF on catalysts.

Table S6. T Calculated Co-O/C=O bond lengths in all initial, transition, and final states.

Figure S1. (a) TEM image and (b) HRTEM image of FCC Co reduced at 873K. The inset image corresponds to FCC Co-associated fast Fourier transformation.

Figure S2. (a) HMF conversion and (b) DMF selectivity over various Co catalysts. Reaction conditions: HMF, 0.378 g; catalyst, 0.05 g; 30 mL THF; agitation speed, 1000 rpm; 453 K; 2 MPa.

Figure S3. Fitting of the pseudo-first-order kinetic model to the hydrogenation of HMF reaction experimental data collected over (a) HCP-Co and (b) FCC-Co.

Figure S4. (a) N_2 adsorption–desorption isotherms, (b) XRD patterns, and (c) enlarged XRD patterns, of HCP-Co/CNTs and FCC-Co/CNTs.

Figure S5. Hydrogenation performance of (a) HCP-Co/CNTs and (b) FCC-Co/CNTs. Reaction conditions: HMF, 0.378 g; catalyst, 0.05 g; 30 mL THF; agitation speed, 1000 rpm; 453 K; 2 MPa.

Figure S6. Cinnamyl aldehyde conversion over HCP-Co and FCC-Co catalysts.

Figure S7. Acetophenone conversion over HCP-Co and FCC-Co catalysts.

Figure S8. Styrene conversion over HCP-Co and FCC-Co catalysts.

Figure S9. Phenol conversion over HCP-Co and FCC-Co catalysts.

Figure S10. Nitrobenzene conversion over HCP-Co and FCC-Co catalysts.

Table S1. Physicochemical properties of Co based catalysts.

Catalysts	Particle size (nm)[a]
HCP-Co	28.2
FCC-Co	32.3
Spent-HCP-Co	30.2

[a] calculated by Scherrer equation

Table S2. Fitting parameters in the pseudo-first-order kinetic analysis of the hydrogenation of HMF over HCP-Co and FCC-Co.

Samples	T (K)	K (min^{-1})	$R^2_{(k)}$	Ea (kJ/mol)	R^2
HCP-Co	413.15	0.015	0.99	30.8	0.99
	433.15	0.029	0.98		
	453.15	0.065	0.99		
	473.15	0.093	0.98		
FCC-Co	413.15	0.0014	0.99	51.2	0.98
	433.15	0.0022	0.98		
	453.15	0.0030	0.99		
	473.15	0.0043	0.98		

Table S3. Physicochemical properties of supported Co catalysts.

Entry	Catalyst	D (nm)	S_{BET} (m^2/g)
1	HCP-Co/CNTs	20.2	133.5
2	FCC-Co/CNTs	17.3	124.5

Table S4. The hydrogenolysis performance of HMF to DMF over different catalysts.

Entry	Catalyst	Condition	HMF Con. (%)	DMF yield (%)	Ref.
1	HCP-Co	180 °C, 2.0 MPa, 2 h, 0.05 g	100	97.7	This work
2	Ru/CoFe-LDO	180 °C, 2.0 MPa, 2 h,	100	98.2	[1]
3	$PdAu_4/GC800$	150 °C, 1.0 MPa, 4 h, 0.05 g	86.8	94.4	[2]
4	Ni/C	180 °C, 4.5 MPa, 2 h, 0.05 g	100	75	[3]
5	$7Ni\text{-}30W_2C/AC$	180 °C, 4.0 MPa, 3h, 0.12 g	100	96	[4]
6	2%Ni-20%Co/C	130 °C, 1.0 MPa, 24h, 0.2 g	99	95	[5]
7	$Cu\text{-}Ni/Al_2O_3$	200 °C, 1.0 MPa, 24h, 0.5 g	99	53	[6]
8	$Cu\text{-}Co/Al_2O_3$	220 °C, 3.0 MPa, 8h, 0.5 g	100	78	[7]
9	$Co@Cu/CoAl_2O_x$	180 °C, 1.5 MPa, 5h, 0.1 g	100	98.5	[8]

Table S5. The adsorption energy (eV) of HMF and BHMF on catalysts.

FCC-Co (111)	E_M	$E_{substrate}$	$E_{M@substrate}$	E_{ads}
HMF	-302.12	-95.07	-398.36	-1.17
HCP-Co (1120)	E_M	$E_{substrate}$	$E_{M@substrate}$	E_{ads}
HMF	-602.24	-95.07	-699.02	-1.71

Table S6. Calculated Co-O/C=O bond lengths in all initial, transition, and final states.

States	HCP-Co (Å)		FCC-Co (Å)	
	C=O (Å)	C=O---Co (Å)	C=O (Å)	C=O---Co (Å)
Initial state (IS)	1.303	1.859	1.343	2.004
Transition state (TS)	1.315	1.864	1.384	1.881
Final state (FS)	1.406	1.814	1.399	1.835

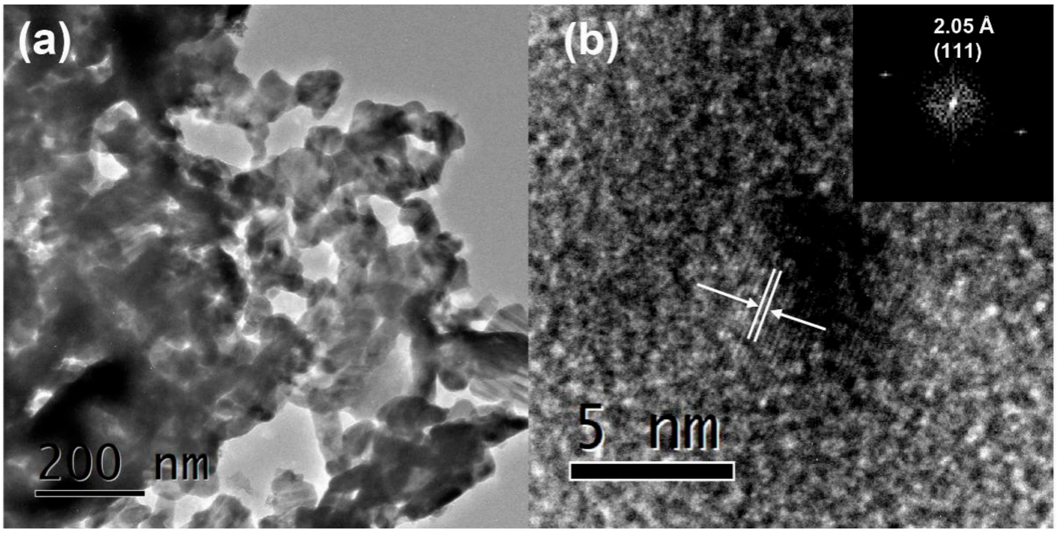

Figure S1. (a) TEM image and (b) HRTEM image of FCC Co reduced at 873K. The inset image corresponds to FCC Co-associated fast Fourier transformation.

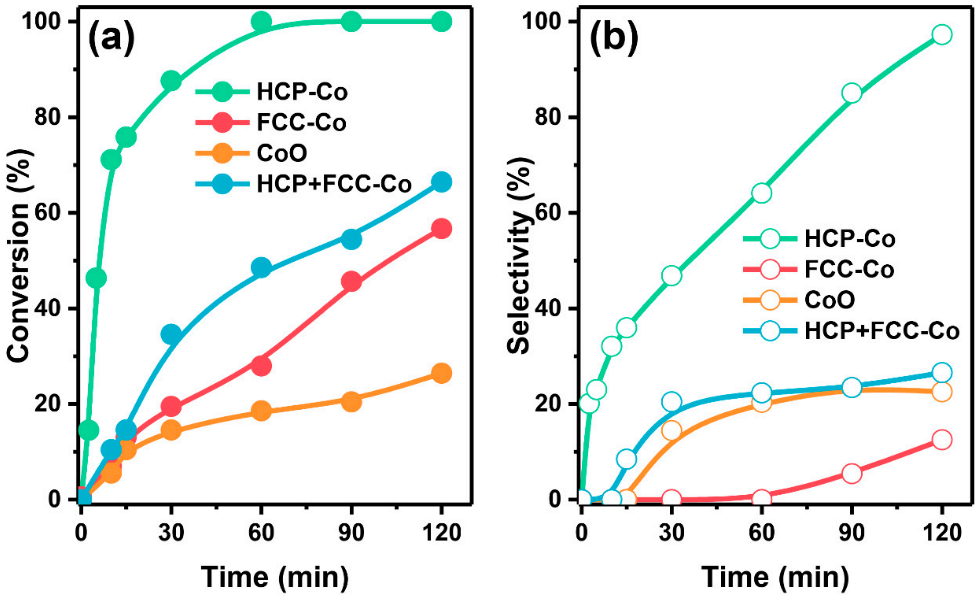

Figure S2. (a) HMF conversion and (b) DMF selectivity over various Co catalysts. Reaction conditions: HMF, 0.378 g; catalyst, 0.05 g; 30 mL THF; agitation speed, 1000 rpm; 453 K; 2 MPa.

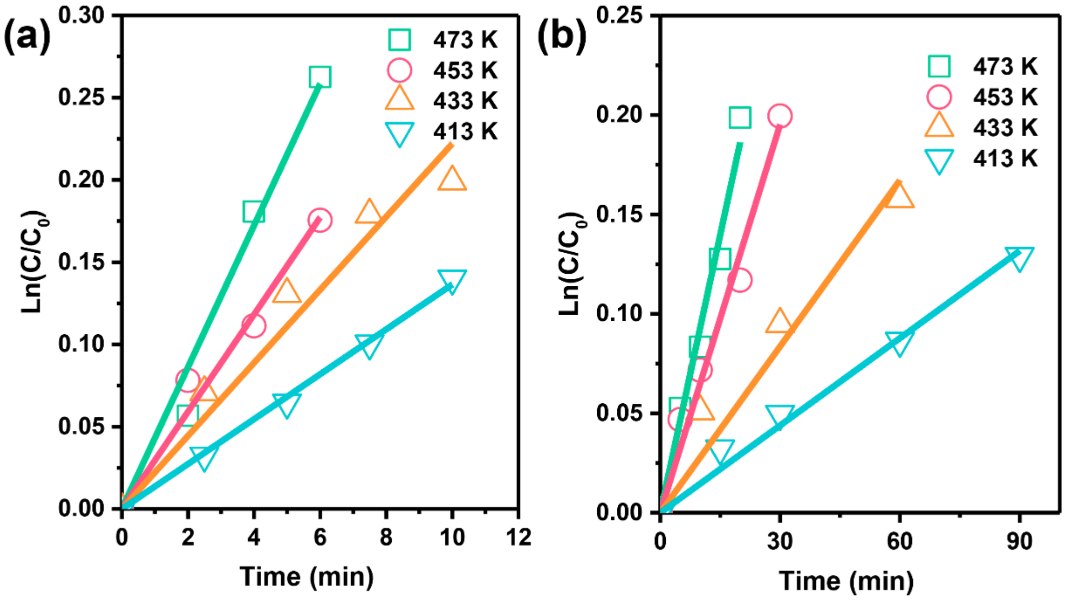

Figure S3. Fitting of the pseudo-first-order kinetic model to the hydrogenation of HMF reaction experimental data collected over (a) HCP-Co and (b) FCC-Co.

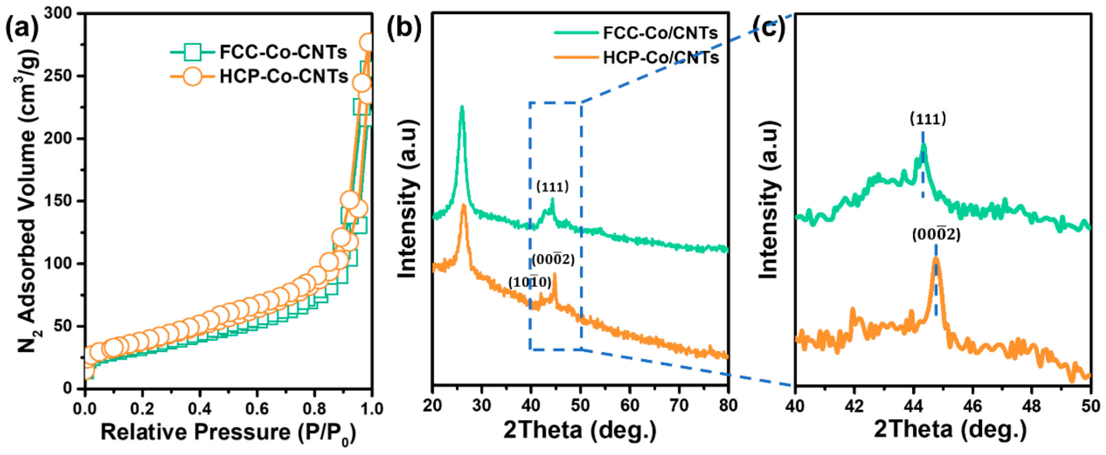

Figure S4. (a) N_2 adsorption–desorption isotherms, (b) XRD patterns, and (c) enlarged XRD patterns, of HCP-Co/CNTs and FCC-Co/CNTs.

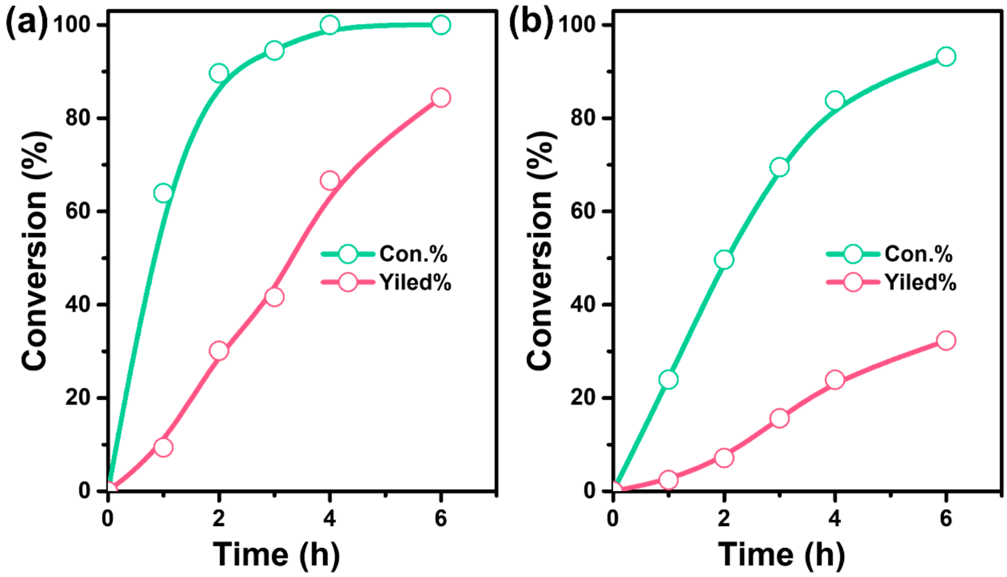

Figure S5. Hydrogenation performance of (a) HCP-Co/CNTs and (b) FCC-Co/CNTs. Reaction conditions: HMF, 0.378 g; catalyst, 0.05 g; 30 mL THF; agitation speed, 1000 rpm; 453 K; 2 MPa.

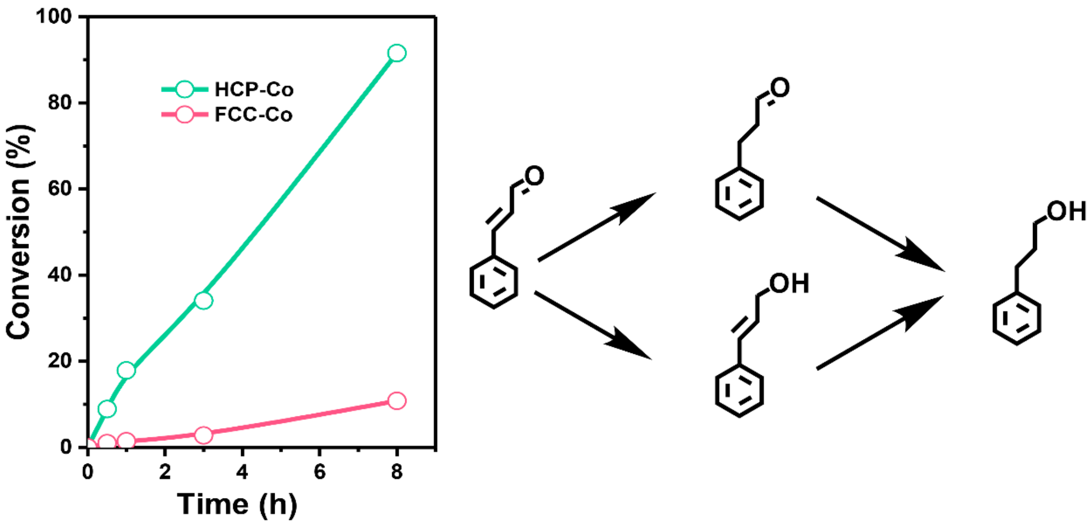

Figure S6. Cinnamyl aldehyde conversion over HCP-Co and FCC-Co catalysts.

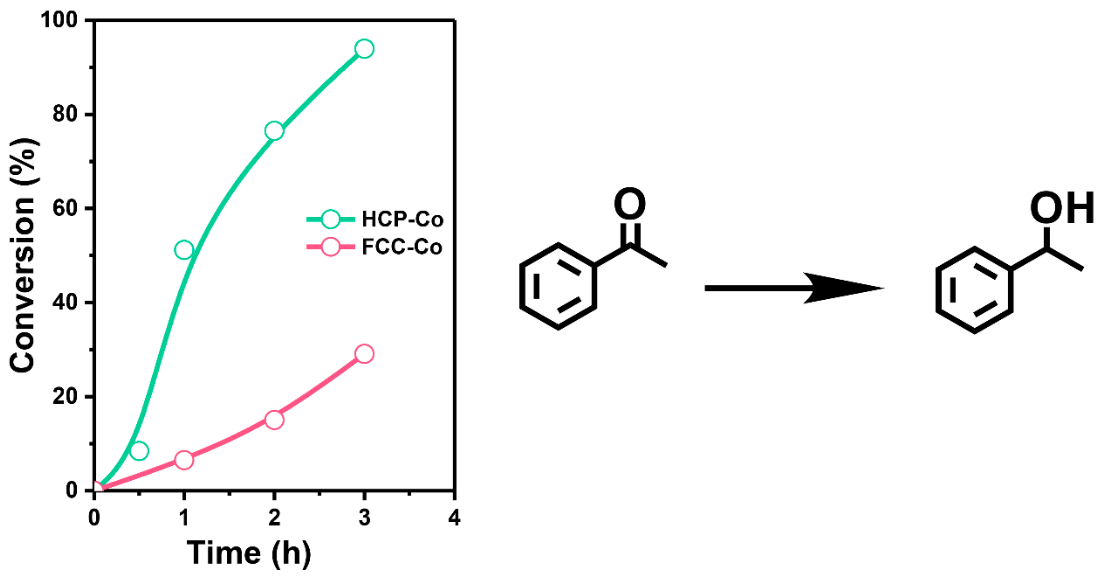

Figure S7. Acetophenone conversion over HCP-Co and FCC-Co catalysts.

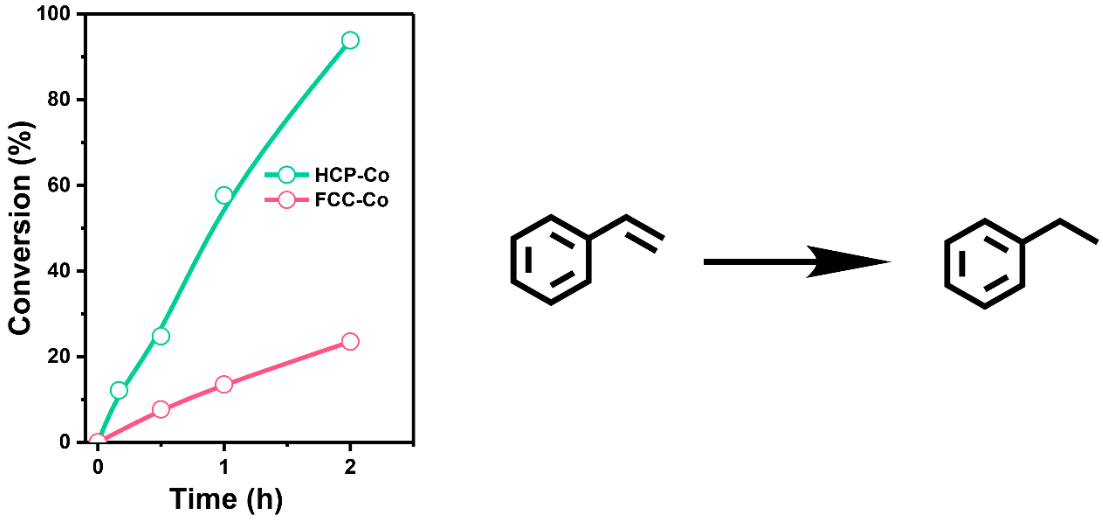

Figure S8. Styrene conversion over HCP-Co and FCC-Co catalysts.

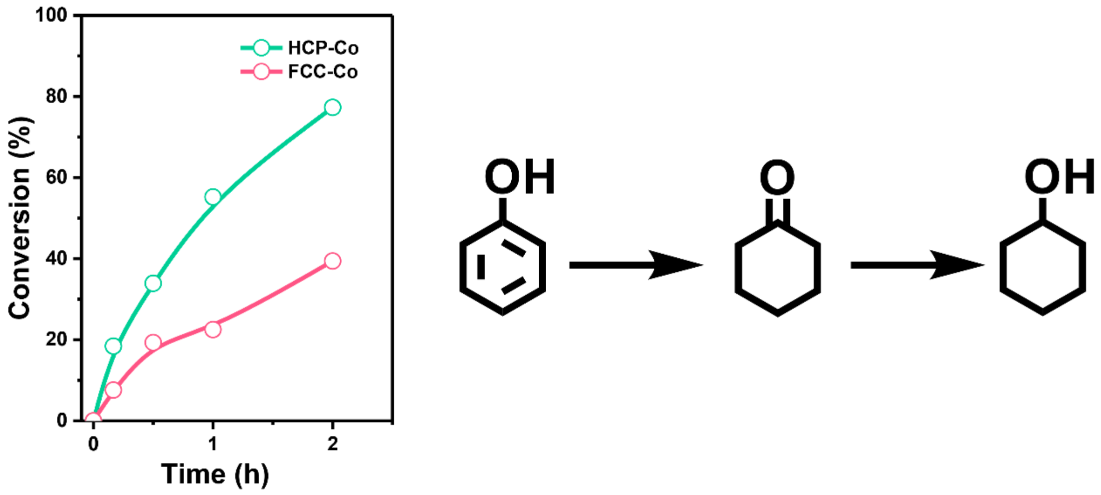

Figure S9. Phenol conversion over HCP-Co and FCC-Co catalysts.

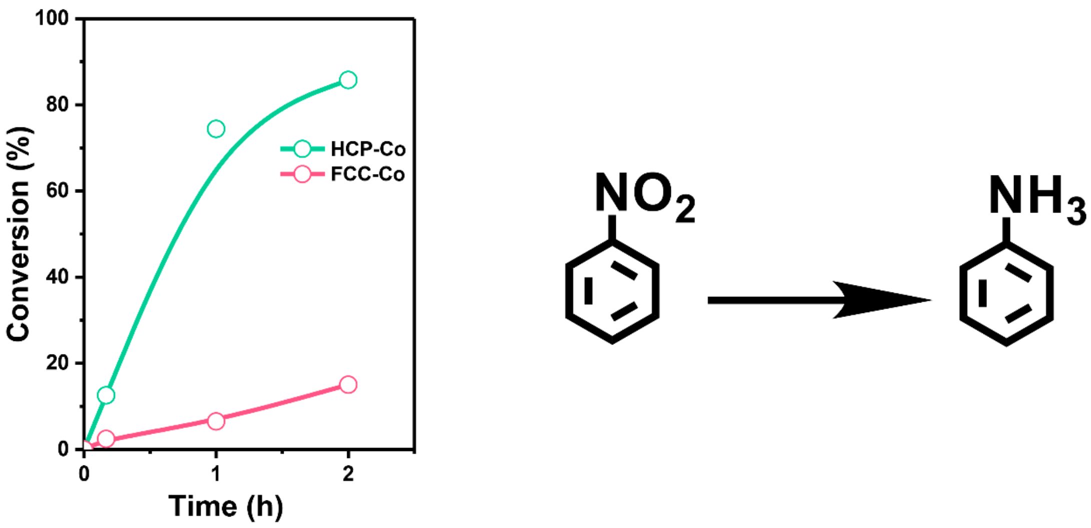

Figure S10. Nitrobenzene conversion over HCP-Co and FCC-Co catalysts.

Reference

1. Li, Q.; Man, P.; Yuan, L.; Zhang, P.; Li, Y.; Ai, S., Ruthenium supported on CoFe layered double oxide for selective hydrogenation of 5-hydroxymethylfurfural. *Mol. Catal.* **2017**, 431, 32-38.
2. Zhang, F.; Liu, Y.; Yuan, F.; Niu, X.; Zhu, Y., Efficient production of the liquid fuel 2,5-dimethylfuran from 5-hydroxymethylfurfural in the absence of acid additive over bimetallic PdAu supported on graphitized carbon. *Energy Fuels* **2017**, 31, 6364-6373.
3. Huang, Y. B.; Chen, M. Y.; Yan, L.; Guo, Q. X.; Fu, Y., Nickel-Tungsten Carbide Catalysts for the Production of 2,5-Dimethylfuran from Biomass- Derived Molecules. *ChemSusChem* **2014**, 7, 1068-1072.
4. Yang, P.; Xia, Q.; Liu, X.; Wang, Y., High-yield production of 2,5-dimethylfuran from 5-hydroxymethylfurfural over carbon supported Ni-Co bimetallic catalyst. *J. Energy Chem.* **2016**, 25, 1015-1020.
5. Srivastava, S.; Jadeja, G. C.; Parikh, J., Influence of supports for selective production of 2,5-dimethylfuran via bimetallic copper-cobalt catalyzed 5-hydroxymethylfurfural hydrogenolysis. *Chin. J. Catal.* **2017**, 38, 699-709.
6. Gyngazova, M. S.; Negahdar, L.; Blumenthal, L. C.; Palkovits, R., Experimental and kinetic analysis of the liquid phase hydrodeoxygenation of 5-hydroxymethylfurfural to 2, 5-dimethylfuran over carbon-supported nickel catalysts. *Chem. Eng. Sci.* **2017**, 173, 455-464.
7. Srivastava, S.; Jadeja, G.; Parikh, J., Synergism studies on alumina-supported copper-nickel catalysts towards furfural and 5-hydroxymethylfurfural hydrogenation. *J. Mol. Catal. A-Chem* **2017**, 426, 244-256.
8. Wang, Q.; Feng, J.; Zheng, L.; Wang, B.; Bi, R.; He, Y.; Liu, H.; Li, D., Interfacial structure-determined reaction pathway and selectivity for 5-(hydroxymethyl) furfural hydrogenation over Cu-based catalysts. *ACS Catal.* **2019**, 10, 1353-1365.
